# Subthalamic nucleus beta and gamma activity is modulated depending on the level of imagined grip force

**DOI:** 10.1016/j.expneurol.2017.03.015

**Published:** 2017-07

**Authors:** Petra Fischer, Alek Pogosyan, Binith Cheeran, Alexander L. Green, Tipu Z. Aziz, Jonathan Hyam, Simon Little, Thomas Foltynie, Patricia Limousin, Ludvic Zrinzo, Marwan Hariz, Michael Samuel, Keyoumars Ashkan, Peter Brown, Huiling Tan

**Affiliations:** a Medical Research Council Brain Network Dynamics Unit at the University of Oxford, OX1 3TH Oxford, United Kingdom; b Nuffield Department of Clinical Neurosciences, John Radcliffe Hospital, University of Oxford, OX3 9DU Oxford, United Kingdom; c Unit of Functional Neurosurgery, Sobell Department of Motor Neuroscience and Movement Disorders, University College London Institute of Neurology, WC1N 3BG London, United Kingdom; d Departments of Neurology and Neurosurgery, King's College Hospital, King's College London, United Kingdom

**Keywords:** STN, Basal ganglia, Force decoding, Motor imagery, Imagined force, Beta suppression, Gamma oscillations, Neuro-feedback, Brain machine interface, Brain computer interface

## Abstract

Motor imagery involves cortical networks similar to those activated by real movements, but the extent to which the basal ganglia are recruited is not yet clear. Gamma and beta oscillations in the subthalamic nucleus (STN) vary with the effort of sustained muscle activity. We recorded local field potentials in Parkinson's disease patients and investigated if similar changes can be observed during imagined gripping at three different ‘forces’. We found that beta activity decreased significantly only for imagined grips at the two stronger force levels. Additionally, gamma power significantly scaled with increasing imagined force. Thus, in combination, these two spectral features can provide information about the intended force of an imaginary grip even in the absence of sensory feedback. Modulations in the two frequency bands during imaginary movement may explain the rehabilitating benefit of motor imagery to improve motor performance. The results also suggest that STN LFPs may provide useful information for brain-machine interfaces.

## Highlights

• We tested to which extent the subthalamic nucleus is involved in motor imagery.
• During real gripping at three force levels beta and gamma activity is scaled.
• Force-dependent modulation also was observed during imagined gripping.
• STN neuro-feedback may support motor training or brain-machine interfaces.

## 1 Introduction

Mental imagery, in addition to physical practice, is known to boost motor performance in comparison to physical practice alone (Avanzino et al., 2009). Two studies have indicated that physical training combined with mental imagery or autogenic training can improve motor performance more than physical exercises alone in patients with Parkinson's disease (Ajimsha et al., 2014, Tamir et al., 2007). Accordingly, it has been suggested that motor imagery exercises might be useful in improving motor control during physical rehabilitation in Parkinson's disease (Abbruzzese et al., 2015). This might be fruitful, as in Parkinson's disease not only motor execution, but also motor planning seems to be impaired (Avanzino et al., 2013, Conson et al., 2014). This idea is corroborated by imaging and transcranial magnetic stimulation studies that have demonstrated abnormal network activity during motor imagery in this patient group (Cunnington et al., 2001, Helmich et al., 2007, Maillet et al., 2015, Rienzo et al., 2014, Thobois et al., 2000, Tremblay et al., 2008). However, the neural basis of the rehabilitating effect of motor imagery in Parkinson's disease is still not known. Better understanding of the network activity underpinning motor imagery might help inform how best to leverage this potential therapeutic adjunct to physical rehabilitation in Parkinsonian patients.

Here we hypothesize that motor imagery involves the basal ganglia in humans in a similar fashion to real movements. We test this by investigating if activity recorded during motor imagery in the basal ganglia is modulated in a task-dependent manner similarly as during real movements. It has already been shown that beta activity decreases in the subthalamic nucleus (STN) local field potential (LFP) during mental imagery of brief wrist extension movements, and that this is not the case during non-motor visual imagery (Kühn et al., 2006). Similar decreases in beta activity have also been reported during passive action observation in the STN (Alegre et al., 2010, Marceglia et al., 2009). But it is still not established whether the extent of such beta changes depends on the intended effort or force of the movement that is imagined. In addition, in motor cortex, mu and beta activity are reduced during motor imagery whereas gamma activity tends to increase, with the latter outperforming changes in mu/beta for decoding of individual imagined finger movements (Liao et al., 2014). Whether gamma activity also increases in the STN during motor imagery is not known. However, there is some reason to suspect that reciprocal changes in beta and gamma activity in the STN might occur during motor imagery and scale with task demands. When patients with Parkinson's disease perform real manual grips at different force levels, beta and gamma activity in the STN are modulated such that the change in the gamma-band subtracted by the change in the beta-band linearly scales with the amount of force applied (Tan et al., 2013). If imagined gripping involves similar network dynamics as real gripping (Jeannerod, 2001), we would predict not only a beta decrease in the STN but also a gamma increase that is amplified with increasing force. Here we test this prediction by analysing local field potential recordings from the STN in Parkinson's disease patients who have undergone deep brain stimulation surgery.

## 2 Materials and methods

### 2.1 Participants

We recorded 11 Parkinson's disease patients who had undergone bilateral implantation of deep brain stimulation (DBS) leads in the STN 2–7 days prior to the recording. Patients underwent DBS surgery to receive chronic high-frequency stimulation of the STN to improve motor symptoms. In the first operation, electrode extension cables were externalized through the scalp to enable recordings. In a second operative procedure, up to 7 days later, a subcutaneous DBS pacemaker was implanted and connected to the electrodes used for chronic stimulation. In this relatively small cohort the number of post-operative days before recording had no obvious effect on the spectral reactivity patterns. The study was approved by the local ethics committee and patients were recorded after obtaining informed written consent. One patient had to be excluded because of excessive movement artefacts during real gripping. This patient had Boston Scientific DB-2201™ leads implanted. Clinical details of all patients included (mean age 61.3 ± 7 years, mean disease duration 9.6 ± 4 years, all right-handed, three female) are listed in Table 1. Recordings were performed in three surgical centres: King's College Hospital and University College Hospital in London and the John Radcliffe hospital in Oxford, UK. For each patient one of the following three macroelectrode models was used: Medtronic 3389 (quadripolar, n = 6), Boston Scientific DB-2201 Vercise (octopolar, n = 2) and Boston Scientific DB-2202 Vercise directional (octopolar, directional, n = 3).

**Table 1:** Clinical details. Age and disease duration are given in years. UPDRS-III: Unified Parkinson's disease rating scale part III. Levodopa equivalent dose was calculated according to Tomlinson et al. (2010). JR = John Radcliffe hospital, KC = King's College Hospital, UCL = University College London Hospital.

ID	Age/sex	UPDRS-III off/on levodopa	Disease duration	Main symptom	Medication (mg/day)	DBS lead	Surgical centre
1	71/m	22/8	12	Tremor	923 mg	Medtronic 3389™	KC, London
2	55/m	27/8	6	Rigidity, gait	1009 mg	Medtronic 3389™	JR, Oxford
3	56/m	17/9	3	Tremor	328 mg	Boston Scientific DB-2201™	KC, London
4	75/m	31/10	11	Gait, tremor	565 mg	Medtronic 3389™	KC, London
5	55/f	84/25	7	Gait, dystonia	1618 mg	Boston Scientific DB-2202™	JR, Oxford
6	62/m	27/4	12	Freezing of gait	955 mg	Medtronic 3389™	KC, London
7	60/m	52/30	8	Freezing of gait,	1282 mg	Medtronic 3389™	UCL, London
8	59/m	53/18	7	Tremor, bradykinesia, dyskinesia	1195 mg	Boston Scientific DB-2202™	JR, Oxford
9	60/f	56/31	14	Tremor, dyskinesia	1750 mg	Medtronic 3389™	KC, London
10	64/f	66/36	16	Rigidity, tremor	1628 mg	Boston Scientific DB-2202™	JR, Oxford

### 2.2 Task

Patients were seated in a comfortable chair with their elbows flexed at about 90°. They held a dynamometer (G200; Biometrics Ltd., Cwmfelinfach, Gwent, UK) in each hand and were asked to grip it with maximal effort three times to obtain the maximum sustainable force before starting the main session. They had to hold the grip for as long as a white dot was presented on a computer screen (4.5 s), and performed this procedure separately for each hand. The time point of the most stable force production was selected manually in each trial and the maximum sustainable force was then computed as the maximum of the three trials.

**Fig. 1:**
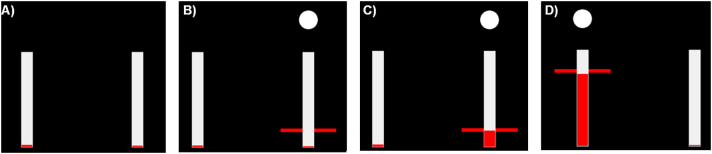
Sequence of visual cues. A) In inter-trial intervals the arms were relaxed and the red cursor bars indicating the grip force registered by the dynamometers were down at the bottom of the white vertical bars. B) At the start of each trial, a white dot and a red horizontal bar appeared either behind the left or the right white bar corresponding to the left and right hand respectively. The cue displayed here instructs patients to grip with their right hand at the lightest level (15% of maximum sustainable force). C) Patients adjusted their grip force such that the red vertical column rises to the same level of the red horizontal bar. D) Example of a trial instructing gripping at the strongest level (85% of maximum sustainable force) with the left hand. In the imagined condition, the red horizontal cues indicate the force level and effector side as during real gripping, however the red vertical bar remained down.

**Fig. 2:**
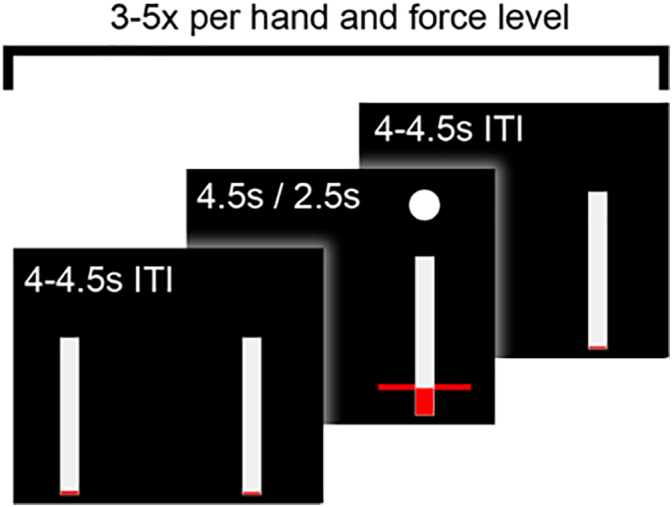
Time course of one block. The inter-trial interval (ITI) was followed by the cue onset (horizontal red bar), which remained present for 4.5 s in the executed gripping condition and for 2.5 s in the imagined gripping condition. Patients relaxed their arms between each trial in the ITI, which varied randomly between 4 and 4.5 s. Each hand and force level was performed 3–5 times depending on the patient's fatigue.

In the first part of the main experiment, patients were presented with a red bar on the screen that instructed them to grip at 15, 50 or 85% of the maximum sustainable force (Fig. 1). The white dot and red bar both appeared either on the left or right field of the screen, which instructed them with which hand they should grip (left or right respectively). These were selected in a pseudo-random order. The red horizontal bars were presented at three different heights, corresponding to the different desired forces. The horizontal red bars were presented for 4.5 s in each trial. The exerted grip force was presented in real time as a vertical red column that increased in proportion to the force delivered. It replaced a vertical white column that corresponded to maximal sustainable force. The inter-trial interval was chosen randomly between 4 and 4.5 s. The time windows and force levels requested were set such that fatigue was kept to a minimum in the context of a time-limited post-operative study. Prior to the first recorded block, patients performed practice trials until they were comfortable with the task. We recorded three blocks in each condition. Each block contained 3–5 trials for each hand and force level (depending on the patient's fatigue, see Fig. 2). After completion of a block, patients were allowed to rest for as long as they wished. This resulted in an overall average number of 11 ± (SD) 2.8 trials per hand and force level.

**Fig. 3:**
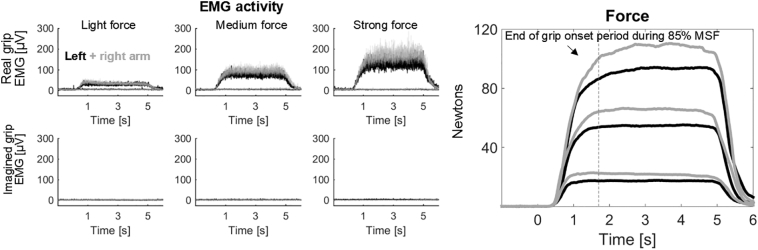
EMG and dynamometer data averaged across patients. Left panels show EMG activity during real gripping (upper row) and imagined gripping (lower row). The three columns show the three different force levels requested by the visual cue. EMG activity at y = 0 in the upper row shows activity of the arm contralateral to the cued one, which was flat and shows that this arm remained relaxed. During imagined gripping no muscle activation was registered. The rightmost plot shows the average force trajectories. The black traces are slightly below the grey ones showing that the maximum force was weaker for the left arm. MSF = Maximum sustainable force.

In the second part of the main experiment, the dynamometers were put aside and patients were asked to rest their arms still on their lap for imagined gripping. Patients were instructed to imagine the gripping action they had just performed without activating any muscles. They were told to keep their arms fully relaxed, and it was pointed out that this would be assessed via recordings from the electromyographic (EMG) electrodes placed on their forearm (Fig. 3). However, they were not provided with real-time visual feedback of EMG or electrophysiological activity. The duration of the cue presentation was shortened to 2.5 s for the imagery condition to make it easier for patients to maintain imagery at the correct level for the whole trial duration and to avoid mind-wandering. Otherwise timings were kept the same as above. The imagery recordings were also split into three blocks allowing for breaks between blocks. Each block contained three trials per hand and force level resulting in an overall average number of 9 ± 1 trials for each hand and force level.

### 2.3 Recordings

Monopolar LFPs were recorded with a TMSi Porti amplifier (2048 Hz sampling rate, common average reference, TMS International, Netherlands) simultaneously with the force data from two dynamometers and two EMG electrodes placed on the left and right extensor muscles of the forearm (data available on request). The data were re-referenced offline to obtain spatially focal bipolar signals by subtracting the data from neighbouring electrode contacts. If single channels were saturated or inactive, the remaining surrounding contacts were used instead.

Subjective performance ratings after each of the three motor imagery blocks were recorded with a questionnaire asking “How well were you able to imagine gripping?” Patients indicated their subjective perception on a visual analogue scale ranging from 0 to 10 with 10 corresponding to “Very well” and 0 to “Not at all”.

### 2.4 Data pre-processing

**Fig. 4:**
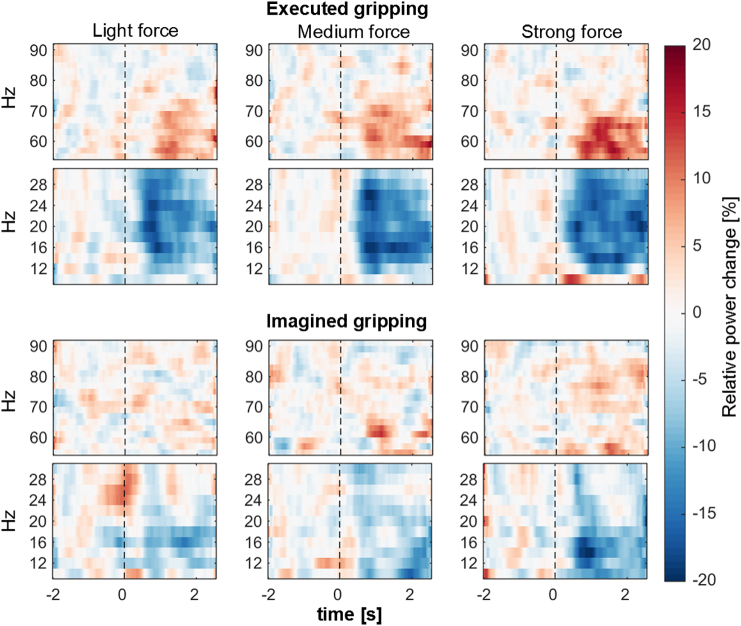
Time-frequency spectrograms during contralateral gripping. Red is a % increase and blue a % decrease relative to baseline, which was the average within − 2:− 0.5 s relative to the onset of the visual cue (shown as the vertical dotted line at t = 0). The three columns are light, medium and strong force levels (from left to right). The upper row of three plots shows gamma (55–90 Hz) and the lower row shows beta (10–30 Hz) power changes in each panel during the task. The upper panel (pair of rows of three plots) is for real gripping and the lower panel shows power changes during motor imagery. Data averaged across 18 STN from 10 patients. To reduce noise in these plots, smoothing was applied for each frequency by computing the average within a 0.5 s moving window.

All data analyses apart from the computation of ANOVAs were performed in MATLAB (v. 2016a, The MathWorks Inc., Natick, Massachusetts). Trials containing artefacts in the force signal or movement artefacts in the LFP signal were removed following visual inspection. Data were down-sampled to 1000 Hz and high-pass filtered with a 5 Hz cut-off (Butterworth filter with a filter order of 6, passed forwards and backwards). Continuous wavelet transform with Morlet wavelets was then applied using the *fieldtrip*-function *ft_freqanalysis* (Oostenveld et al., 2011). The wavelet width was set to 8 cycles for frequencies below 30 Hz and to 26 cycles for frequencies higher than 50 Hz. For the gamma-band, power was averaged between 55 and 85 Hz. This range was selected to avoid line interference and to capture the reactivity observed particularly in this range (Fig. 4; results were highly similar if statistics were computed with a 55–81 Hz band). 26 cycles thus provided an estimate of power within a window of about 0.37 s, which was similar to the 0.40s window including 8 cycles at 20 Hz.

In the next step, the power of the resulting time-frequency decomposition was down-sampled to 50 Hz and baseline-normalized. After computing the median across trials, a window ranging from − 2 to − 0.5 s prior to cue onset was chosen as baseline (− 0.5 s to limit inclusion of any preparatory beta decrease that might have occurred close to the cue). We analysed only bipolar contacts that showed a significant movement-related beta decrease upon real gripping across all contralateral grip trials irrespective of force level (assessed by one-sample *t*-tests against zero) and thus should be located close to the dorsal, i.e. sensorimotor region, of the STN (Levy et al., 2002, Weinberger et al., 2006, Williams et al., 2005). From these bipolar contacts, the bipolar contact and the frequency (± 2 Hz) with the largest beta decrease was selected. If none of the bipolar contacts of an electrode recorded a significant beta decrease, this electrode was excluded from further analyses. In total, 1 of 20 electrodes was excluded.

To assess the spectral change, we tested the time-averaged normalized power from 0.5 to 2.5 s after the cue for frequencies in the beta range (12–30 Hz in 2 Hz steps). The window started at 0.5 s to allow for reaction times, which were around 0.5 s as displayed in Fig. 3. EMG activity was filtered between 3 and 400 Hz (Butterworth filter with a filter order of 6, passed forwards and backwards) and rectified. To examine if the velocity of grip onset differed between force levels, the peak rate of force generation was calculated as the maximum of the differentiated force.

### 2.5 Statistical analyses

Gripping consists of an onset phase and a later period during which grip force is more or less sustained. Thus, we separated the data into an early and late time window. Fig. 3 shows that movement started only about 0.5 s after the cue and that it took up to 1.7 s after the cue to reach the desired level across the different cued grip strengths. Thus, the early window was defined to span 0.5–1.7 s and the late window 1.7–2.5 s. As durations of executed and imagined movements have been reported to be similar (Papaxanthis et al., 2002), we assumed that these windows adjusted to the time course observed during real gripping reflected similar time periods related to the motor process in the imagined condition.

For each subject, the median power change across trials was computed for each force level to obtain a robust estimate of power changes, and then this was averaged within the two time windows. The data were then subjected to the following repeated-measures ANOVA using SPSS (v. 22, IBM SPSS Statistics for Windows, Armonk, NY: IBM Corp.):

A 2 (*task*: real and imagined gripping) ∗ 2 (*effector side*: contra- and ipsilateral grips) ∗ 3 (*force level*: low, medium and high force) ANOVA with the gamma-beta power changes (Tan et al., 2013) as dependent variable to test for significant differences across force levels and to see if the degree of modulation was lateralized. We then examined within-subject contrasts to test if the linear component of the factor *levels* was also significant with beta or gamma modulation alone as dependent variable. Greenhouse-Geisser correction was applied where necessary and the correction factor ɛ reported if the sphericity assumption was violated. Post-hoc pairwise comparisons were performed with *t*-tests or Wilcoxon signed-rank tests if the normality assumption was violated. To test if the linear component of the factor levels was significant, we computed within-subject contrasts in SPSS.

Correlations of power changes (beta, gamma and gamma-beta power changes) between the real and imagined gripping at the strongest force level were computed as Spearman's rank correlation coefficients with bootstrapped confidence intervals using the *Spearman* function from the Robust correlation toolbox (Pernet et al., 2013). Finally, we also tested if the gamma-beta power change from the lightest to the strongest level during motor imagery was correlated with subjects' self-rating of how well they were able to imagine gripping (again using the *Spearman* function).

## 3 Results

### 3.1 Behavioural data

Force and EMG trajectories show that patients were able to adjust their grip force according to the cues provided during real gripping (Fig. 3). The average maximum sustainable force was 132 ± (SD) 69 N for the right and 121 ± 54 N for the left hand. The force applied in the sustained period (1.7–2.5 s) during low, medium and high force trials was on average 17 ± 3%, 49 ± 4% and 81 ± 8% of the maximum sustainable force. A 2 × 3 ANOVA with peak velocity of the force onset as dependent variable and *side* (left, right) and *force levels* as factor showed that only the factor *force level* was significant (*F*_*2,18*_ = 6.1, *P* = 0.010), and not the factor *side* (*P* = 0.083) nor the interaction (*P* = 0.780). Peak rate of force generation during light grips differed significantly from the peak velocity of medium (*P* = 0.002) and strong (*P* = 0.002) grips, whereas peak rate of force generation did not differ significantly between medium and strong grips (*P* = 0.084). The EMG activity of the arm contralateral to the cued one was flat showing that muscles in this arm were not co-activated (Fig. 3).

In the imagined condition, no EMG activity was visible on either side, confirming that patients kept their arms relaxed as instructed (Fig. 3, bottom row). Patients' subjective rating of how well they were able to imagine gripping was on average 6.6 ± 1.3 on a scale from 0 to 10, with 0 meaning “Not at all” and 10 meaning “Very well”.

### 3.2 Contact and frequency band selection

**Fig. 5:**
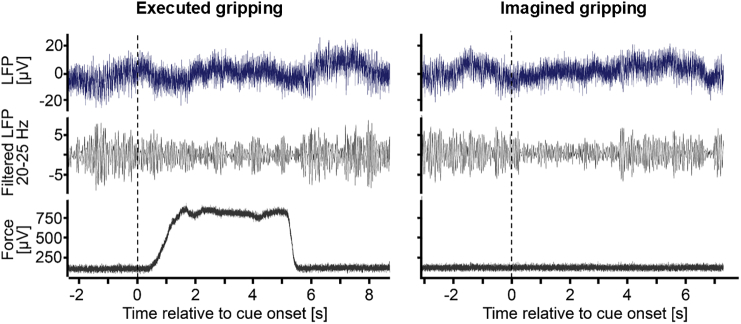
LFP recording from one representative patient. The first row shows the raw bipolar local field potential (2 s drift correction removal applied), the second row shows the beta oscillations within the same data filtered between 20 and 25 Hz and the third row shows the force trajectory recorded with the dynamometer. The left panel shows one trial of gripping with the contralateral hand and strongest force (4.5 s cue duration, onset at 0 s). The right panel shows the same channel in a trial prompting imagination of gripping with the same hand and same force level (2.5 s cue duration).

In one patient, beta was significantly modulated only in the right electrode, and in another patient the maximum sustainable force was set too low for one side because of a technical error and thus was discarded. In total, 18 STN from 10 patients were included for analysis of the LFP data. The average beta frequency with the highest movement-related reactivity was 22 ± 6 Hz. Fig. 5 shows LFP recordings from one patient performing a representative trial of executed and of imagined gripping at the strongest force level. The 20–25 Hz filtered beta oscillations decrease clearly with cue onset.

### 3.3 Gamma-beta power changes depend on the force level

To assess if the observed power changes (Fig. 4) were modulated in a force-dependent manner, and if these changes were lateralized, we performed a 2 (*task*: real and imagined gripping) ∗ 2 (*effector side*: contra- and ipsilateral grips) ∗ 3 (*force level*: low, medium and high force) ANOVA with the gamma-beta power changes as the dependent variable (see Tan et al., 2013).

**Fig. 6:**
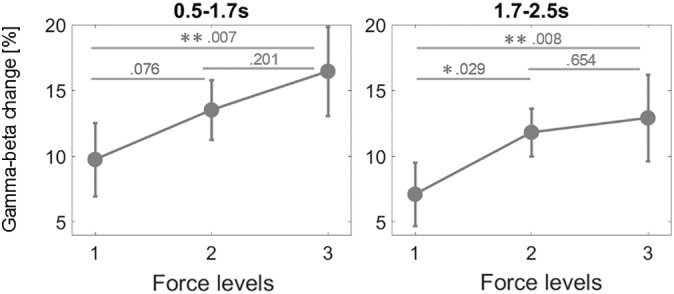
Force-dependent changes of gamma-beta activity averaged across task and effector side. Force-dependent contralateral power changes are separately displayed for each task in this figure. In both the early and late time window force increases with force level. The difference between power at the highest and lowest force level is significant in both windows. * denotes that power significantly differed between levels after controlling for multiple comparisons with FDR-correction (*< 0.05, **< 0.01).

All three main effects were significant for both the early (*task*: *F*_1, 17_ = 13.8, ɛ = 1.0, *P* = 0.002; *effector side*: *F*_1, 17_ = 28.8, ɛ = 1.0, *P* < 0.001; *force level*: *F*_2, 34_ = 5.0, ɛ = 0.98, *P* = 0.013) and the late time window (*task*: *F*_1, 17_ = 14.1, ɛ = 1.0, *P* = 0.002; *effector side*: *F*_1, 17_ = 37.9, ɛ = 1.0, *P* < 0.001; *force level*: *F*_2, 34_ = 4.3, ɛ = 0.92, *P* = 0.026). Post-hoc pairwise comparisons between force levels are shown in Fig. 6. Within-subject contrasts were also computed to test if the linear component of the factor levels was significant, which was confirmed again for both the early and the late time window (Table 2).

**Table 2:** Linear within-subject contrasts of the factor *levels*. The F-statistic, which tests if power changes were linearly related to the increasing force levels, was significant and highest for the combined change in gamma - beta.

Power	Early window	Late window
Gamma - beta	*F*_1, 17_ = 9.4, *P* = 0.007	*F*_1, 17_ = 9.0, *P* = 0.008
Beta only	*F*_1, 17_ = 6.3, *P* = 0.023	*F*_1, 17_ = 6.2, *P* = 0.024
Gamma only	*F*_1, 17_ = 5.9, *P* = 0.027	*F*_1, 17_ = 5.0, *P* = 0.040

**Fig. 7:**
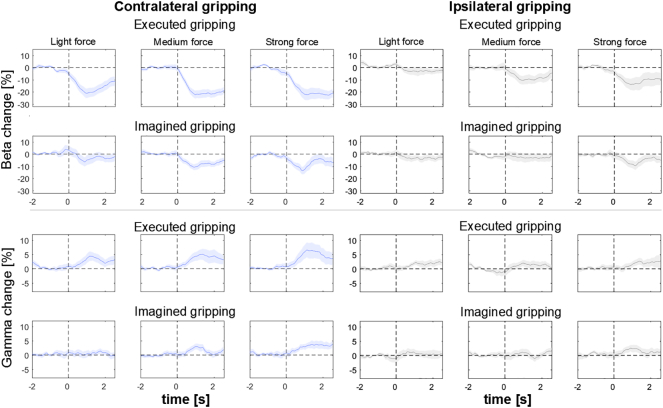
Subject-averaged power changes (median across trials). The upper and lower rows show beta and gamma power changes, respectively. The left column shows contralateral grips and the right column ipsilateral grips. Onset of the visual cue is shown as the vertical dotted line at t = 0. To reduce noise, smoothing was applied by computing the average within a 1 s moving window for this visualization. Shaded regions denote standard error of the mean.

In addition to the main effect, we found a significant interaction of *effector side* ∗ *task* in both the early (*F*_1, 17_ = 6.4, ɛ = 1.0, *P* = 0.022) and late window (*F*_1, 17_ = 5.2, ɛ = 1.0, *P* = 0.037). Post-hoc pairwise comparisons for both windows showed that power differed between contra- and ipsilateral grips only significantly in the real gripping condition (*early window*: realContra = 26.0 ± 17.9, realIpsi = 10.9 ± 15.4, *P* < 0.001; *late window*: realContra = 22.6 ± 14.3, realIpsi = 8.7 ± 15.0, *P* < 0.001) and not in the imagined condition (*early window*: imaginedContra = 9.9 ± 10.1, imaginedIpsi = 6.2 ± 10.3, *P* = 0.111; *late window*: imaginedContra = 7.6 ± 9.6, imaginedIpsi = 3.5 ± 10.4, *P* = 0.063). The beta and gamma power traces in Fig. 7 also depict this difference over time.

None of the other interactions were significant (*early window*: effector side ∗ force level *P* = 0.503, task ∗ force level *P* = 0.835, effector side ∗ task ∗ force level *P* = 0.231; *late window*: effector side ∗ force level *P* = 0.360, task ∗ force level *P* = 0.291, effector side ∗ task ∗ force level *P* = 0.976). Even though the relative power modulation was less in the imagined condition than during real gripping, as confirmed by the main effect of task, the fact that the interaction *task* ∗ *force level* was not significant indicates that the force-dependent modulation of power observed during real gripping resembled that during imagined gripping.

We also tested if the individual frequency bands, i.e. gamma or beta alone would result in a similar or possibly larger F-statistic for the linear component of the within-subject contrast of levels. The linear component was significant in the late window for both cases, but the F-statistic was reduced indicating that the combination of the two features was superior in detecting a linear relationship (Table 2).

**Fig. 8:**
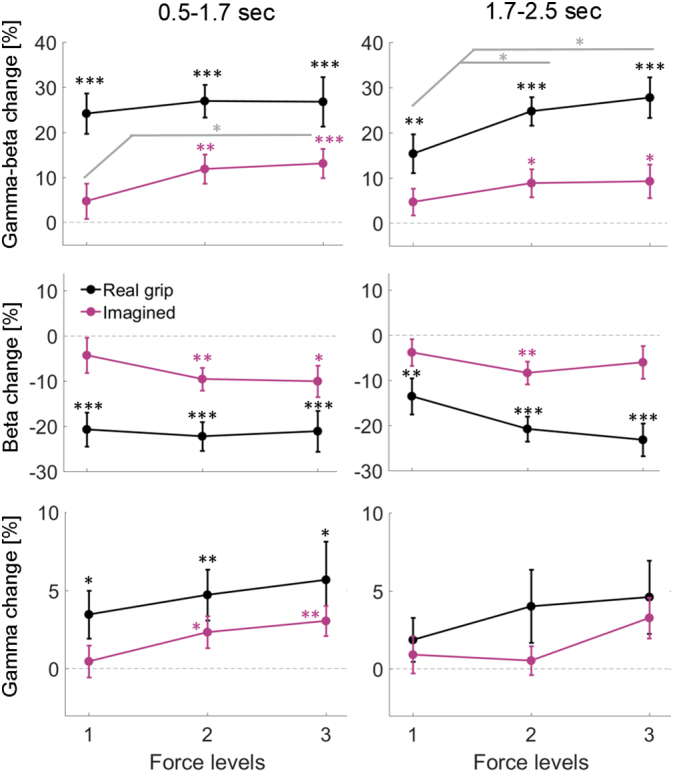
Mean power across patients for the three force levels in the real and imagined gripping condition. * above markers denote that beta ERD was significantly different from zero (*< 0.05, **< 0.01, ***< 0.001, FDR-correction was applied in each plot for the 6 multiple comparisons, grey * did not survive FDR-correction).

It has not yet been shown whether gamma activity in the STN is modulated during motor imagery. Thus we computed pairwise comparisons between the three combinations of force levels for the gamma and beta-band separately for the two tasks. Fig. 8 shows how combined gamma-beta power (top row), and how beta and gamma power separately change across the three different force levels in the two conditions. Beta activity in the sustained period (and in the early window during motor imagery) differed most strongly between the lowest level and the highest two force levels, whereas for gamma activity the increase seemed to be more linear. Such a floor effect of the beta decrease at medium force levels has previously been reported during real gripping (Tan et al., 2013). Gamma activity was significantly increased during imagination of gripping at the highest force level, particularly in the early time window. The average increase in the late time window did not survive FDR-correction.

**Fig. 9:**
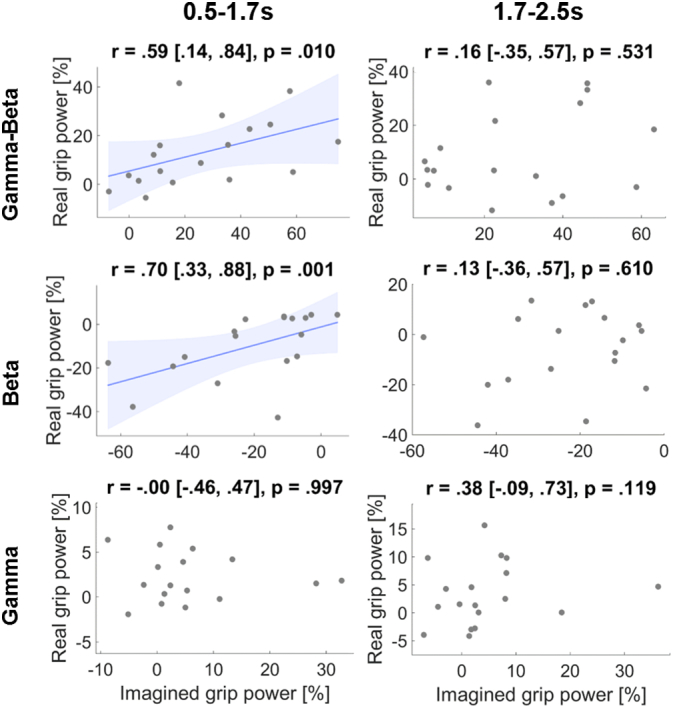
Correlations of power changes between the imagined and real grip condition. Each point denotes one recorded STN displaying its power change in the imagined (x-axis) and real grip condition (y-axis). Correlations were significant only for beta power and the combination of gamma-beta power taken from the early window (during grip onset). Plot titles denote Spearman's rho followed by its 95% bootstrapped confidence intervals and *P*-value. Lines were fitted with a least squares linear regression and shaded areas denote 95% confidence intervals.

To examine if reactivity in one task was related to reactivity in the other condition, we also computed correlations between power changes during gripping and imagination at the highest force level. This correlation was significant for gamma-beta and for beta alone in the early time window (Fig. 9).

We also tested if the amount of power reactivity during imagined gripping was correlated with patients' self-rating of how well they were able to imagine gripping. We correlated the relative power change from the light to the strong force level (averaged across both STN) with the self-rating (averaged across individual ratings following each block). The correlation was positive but it was not significant (Spearman's rho = 0.41, 95% CI = [− 0.39, 0.89], *P* = 0.244).

## 4 Discussion

We found that STN LFP activity was modulated by the intended force during imagined gripping, suggesting that the STN LFP carries information about intended force generation even in the absence of actual muscle activity or movement-related peripheral proprioceptive feedback. In addition, to our knowledge, this is the first report of increased STN gamma activity during motor imagery, and shows that the increase of gamma activity in the STN is not only associated with actual movement or muscle activation. The spectral changes identified during imagined movements further strengthen the idea that these changes relate to motor effort rather than force coding per se (Tan et al., 2015). This signal may then be transcribed into force through the motor cortex and related effectors. The evidence that STN LFP activity is modulated and linearly scaled with planned force during motor imagination also helps to motivate the exploration of the role of motor imagination in promoting the effects of physical rehabilitation (Abbruzzese et al., 2015). In addition, the results suggest that similar spectral reactivity might be retained within the STN in patients who cannot move and therefore have no movement-related sensory feedback. This is important, as it may mean that the STN LFP could provide an ancillary brain-machine-interface signal for graded response control, such as grasp force control, in paralysed patients.

Although within-subject contrasts confirmed linear scaling of spectral changes during early and later phases of real and imaginary gripping, the average reactivity of beta and gamma oscillations was less in the imagined condition than during real gripping. This may have been due to several reasons. Firstly, patients may have been inattentive or failed to sustain the imagined grip in spite of the short duration of the imagination blocks. Secondly, although sensory reafference may not be necessary for modulating oscillations it may act to reinforce any modulation. Finally, if changes in beta and gamma activity reflect effort as previously suggested (Tan et al., 2015), then the reduced modulation might also reflect reduced effort during imagined gripping, assuming that motor imagery might present the motor network with fewer demands than real movement.

We also found that beta-gamma reactivity during imagined gripping at the highest force level significantly correlated with reactivity during real gripping, in the early time window at least. This predominately originated from a relationship within the beta-band. A similar relationship between beta activity modulation during real and imagined movement has also been reported previously in another motor imagery task (Kühn et al., 2006). The correlation provides some evidence that the spectral reactivity during real and imagined tasks comes from a similar generator within the subthalamic area, perhaps the dorsolateral ‘motor’ STN. Our study thus provides further evidence that imagined and real movements may share common pathways (Gerardin et al., 2000).

### 4.1 Study limitations

It should be acknowledged that the observed spectral modulation may not necessarily reflect normal physiological activity as we recorded from patients with Parkinson's disease. However, patients were recorded on dopaminergic medication to minimize pathological activity linked to depletion of dopamine (Weinberger et al., 2006) and vividness of motor imagery seems to be preserved in comparison to age-matched controls even after medication withdrawal (Peterson et al., 2012).

Even though we found that gamma-beta activity during real and imagined gripping at ~ 15% of the maximum sustainable force differed from gripping at ~ 50 or ~ 85%, the latter two levels were not clearly distinguishable in terms of power changes. Moreover, the beta decrease during onset of real gripping seemed to be saturated as it was similar across all three different levels of force. These findings differ from those in our previous report of real gripping (Tan et al., 2013), and may be related to the fact that in the current study the peak rate of force development only differed between the lowest level and the medium or highest force levels but not between the latter two. In addition, Tan et al.'s study had no visual cues and thus task demands may have differed slightly considering that the initial effort of matching grip force to a visual cue may be similarly high for light and strong grips. Also, power changes were aligned to grip onset in their analysis instead of cue onset as in our study, which was necessary for comparing executed and imagined movements lacking a measurable grip onset.

Although we monitored muscle activity of the arm and closely watched if patients sat still during the task, we cannot entirely rule out subtle contractions of other muscle groups. However, this is a general problem in BCI control and can only ever be tested with certainty in patients who are completely locked-in.

### 4.2 Translational significance of the findings

Beta and gamma activities were not only modulated during imagined gripping of the contralateral, but also of the ipsilateral hand. Significant lateralization only occurred during real gripping. Thus, although the STN LFP signal might provide a potential substrate for force decoding in applications involving brain machine interfaces, it may not provide sufficient information for the selection of the limb prosthesis to be activated. Accordingly, selection of an effector might require the consideration of additional signals, such as from the cerebral cortex, as motor cortex also seems to carry, at least to some extent, information about imagined force levels: Cortico-spinal excitability has been found to differ between low and high imagined force levels with higher excitability at higher forces (Helm et al., 2015). In addition, cortical activity during imagery of sustained grasping at different force levels seems to be distinguishable from activity during rest and during imagined alternating movements (Murphy et al., 2016, Yin et al., 2015). Yet it seems difficult to discriminate between different imagined force levels from cortical recordings alone (Murphy et al., 2016, Zaepffel et al., 2013). To our knowledge only one study has successfully extracted features that carry information about imagined force from scalp EEG (Fu et al., 2014). Feature extraction was based only on low-pass filtered data and the contribution of beta or gamma activity was not examined.

Our findings suggest that the combination of gamma-beta is better suited for distinguishing different force intentions than either band in isolation for both real and imagined actions (see also Tan et al., 2013). Our results also indicate that force levels can be differentiated best in the early phase of movement imagination, raising the possibility that STN signals during imagined or fictive gripping of different strengths could potentially provide a relatively fast and dynamic substrate for the control of neuroprostheses, and in particular communication devices, in patients who otherwise cannot move. The only successful approach for communicating with completely locked-in patients to date used functional near infrared spectroscopy to measure hemodynamic changes over sensorimotor and temporal or frontocentral cortex (Chaudhary et al., 2017, Chaudhary et al., 2016, Gallegos-Ayala et al., 2014). However, this communication technique would still benefit from improved decoding accuracy and speed as it depends on relatively slow changes in oxygenation levels.

Another potential, related application is to combine neuro-feedback and motor imagery to help improve motor symptoms in Parkinson's disease (Subramanian et al., 2011). To this end it would be interesting to explore if gamma and/or beta modulation can be intentionally enhanced by repeated practice of mental imagery with concurrent feedback. Importantly, the improvement in motor symptoms in Parkinson's disease after levodopa administration has been linked to a decrease in beta and an increase in gamma activity at the level of the basal ganglia (Brown et al., 2001, Williams et al., 2002). Improved modulation of these oscillations through combined motor imagery and neuro-feedback training might thus facilitate symptom improvement, which has been reported after combined motor and mental imagery exercises in patients with Parkinson's disease (Ajimsha et al., 2014, Tamir et al., 2007).

Taken together, beta and gamma modulation in the STN may help encode motor effort rather than executed force per se. Moreover, this study suggests that spectral changes in STN activity are not dependent on peripheral feedback, opening up the possibility that STN LFP signals might provide a substrate for effort and hence force decoding in the control of neuroprosthetic devices. Finally, the very fact that STN activity is modulated during motor imagery helps motivate further studies of the latter as adjunctive therapy in the physical rehabilitation of Parkinson's disease (Ajimsha et al., 2014, Tamir et al., 2007, Abbruzzese et al., 2015) and of the use of signals from the STN as a basis for neurofeedback training.

## Author contributions

PF, AP, PB and HT designed the study. BC, ALG, TZA, JH, SL, TF, PL, LZ, MH, MS and KA performed DBS surgeries and helped with patient recruitment. PF and HT acquired the data. PF analysed the data under supervision of PB and HT. BC assessed anatomical electrode locations. All authors contributed in writing and revising the paper.

## Competing financial interests

BC has received travel support and unrestricted educational grants for organising CPD events from Medtronic, St. Jude Medical and Boston Scientific (manufacturers of DBS electrodes). TA has performed consultancy for and received speaking fees from Medtronic. SL has been a participant in a DBS teaching course funded by Medtronic, the manufacturer of the electrodes used in this study. TF, PL and LZ have received speaking fees and travel support from Medtronic and St. Jude Medical. PB has received fees and non-financial support from Medtronic and personal fees from Boston Scientific.
